# NEUROSCIENCE AND AGING

**DOI:** 10.1093/geroni/igad104.0729

**Published:** 2023-12-21

**Authors:** Gregorio Valdez

## Abstract

In this symposium, experts will highlight discoveries that are beginning to bridge the gap between basic neuroscience research and development of therapies to mitigate the ravages of normal aging and age-associated neurological diseases on the nervous system. The symposium will cover cellular, molecular and behavioral approaches to researching aging of the nervous system. It will provide insights about how aging impacts the fate of neurons and their synapses and how age-related changes in cellular maintenance, molecular signaling, and glial function ultimately diminish cognitive and motor function. Dr. Miranda Orr will provide insights about the contribution of senescent cells to age- and disease-associated degeneration of the nervous system. Dr. Julie Andersen will present on how cells in the nervous system use their own organelles to repair damages to avoid dysfunction during aging and in Alzheimer’s Disease. Dr. Gregorio Valdez will review recent discoveries revealing the initial cellular and molecular changes that impair voluntary movements during aging. Dr. Scott Pletcher will present evidence that cellular and molecular processes important for feeding, mating and avoiding danger in flies also play key roles in healthy aging of the nervous system. The information provided by the speakers will foster discussion among all attendees about the challenges and opportunities to accelerate translation of discoveries to protect the nervous system from aging and associated diseases.

